# Cardiovascular Outcomes of Perioperative Sodium‐Glucose Transporter 2 Inhibition in Cardiac Surgery Patients: An Open‐Label Randomized Pilot Study

**DOI:** 10.1111/aas.70075

**Published:** 2025-06-22

**Authors:** Lars I. P. Snel, Maartina J. P. Oosterom‐Eijmael, Yugeesh R. Lankadeva, Mark P. Plummer, Benedikt Preckel, Coert J. Zuurbier, Jeroen Hermanides, Daniel H. van Raalte, Abraham H. Hulst

**Affiliations:** ^1^ Department of Anesthesiology Amsterdam University Medical Center Amsterdam the Netherlands; ^2^ Department of Endocrinology Amsterdam University Medical Center Amsterdam the Netherlands; ^3^ Amsterdam Cardiovascular Sciences Research Institute Amsterdam the Netherlands; ^4^ Amsterdam Gastroenterology Endocrinology Metabolism Research Institute Amsterdam the Netherlands; ^5^ Preclinical Critical Care Unit, Florey Institute of Neuroscience and Mental Health The University of Melbourne Melbourne Victoria Australia; ^6^ Department of Critical Care, Melbourne Medical School The University of Melbourne Melbourne Victoria Australia; ^7^ Department of Anesthesia Austin Health Melbourne Victoria Australia; ^8^ Intensive Care Unit, Royal Adelaide Hospital Adelaide South Australia Australia; ^9^ School of Medicine University of Adelaide Adelaide South Australia Australia; ^10^ Amsterdam Public Health Research Institute, Quality of Care Amsterdam the Netherlands; ^11^ Laboratory of Experimental Intensive Care and Anesthesiology, Amsterdam UMC University of Amsterdam Amsterdam the Netherlands

## Abstract

**Background:**

Previous studies have shown cardiovascular benefits of SGLT2 inhibitors. The aim of this study was to evaluate the cardiovascular effects of perioperative SGLT2 inhibition in patients undergoing cardiac surgery.

**Methods:**

In this open‐label pilot study, adult patients undergoing cardiac surgery were randomized to receive a daily dose of empagliflozin (10 mg; oral) 3 days before surgery until 2 days after surgery, or standard of care. Blood pressure, heart rate, postoperative diuresis, intravenous fluid administration, fluid balance, and vasoactive support were compared between groups during the first 24 postoperative hours.

**Results:**

About 55 patients (sex: 73% male, age: 66 ± 10 years, BMI: 28 ± 4 kg/m^2^, empagliflozin *n* = 25, control *n* = 30) were included in this study and analyzed according to the intention‐to‐treat principle. Empagliflozin was associated with increased diuresis, mean difference 549 mL (95% CI 258–839, *p* < 0.001), and less positive fluid balance postoperatively, mean difference −1217 mL (95% CI −2373– −61, *p* = 0.039). Empagliflozin did not increase the amount of intravenous fluid administered. In the empagliflozin group, norepinephrine was infused for 11.8 ± 11.5 h compared to 19.3 ± 19.3 h in the control group (*p* = 0.080). No significant between‐group differences were observed in postoperative blood pressure and heart rate.

**Conclusions:**

Perioperative SGLT2 inhibition was associated with increased diuresis and lesser fluid accumulation without an increase in vasopressor requirement. These data warrant validation and further evaluation in a larger‐scale, double‐blind, placebo‐controlled trial.

**Editorial Comment:**

In this sub‐study of the randomized MERCURI‐2 trial of perioperative empagliflozin for nondiabetics in cardiac surgery, the authors describe the hemodynamic outcomes and fluid status of the patients. The authors noted a higher urine output and a more negative fluid balance in the intervention group compared to the placebo group. An interesting observation is the trend towards lower noradrenaline usage, although this cannot be concluded with confidence based on this data. The findings support considering and further studying the use of these medications for patients with cardiovascular disease undergoing surgery.

**Trial Registration:** https://onderzoekmetmensen.nl/en/trial/26563 Identifier: NL9561

## 1 Introduction

Sodium‐glucose transporter‐2 (SGLT2) inhibitors are widely prescribed for patients with heart failure (HF) due to their beneficial cardiovascular effects [1, 2, 3]. In large cardiovascular outcome trials and studies in people with HF with preserved ejection fraction (HFpEF) and HF with reduced ejection fraction (HFrEF), SGLT2 inhibitors reduced heart failure symptoms as well as heart failure hospitalizations and cardiovascular death [2, 3, 4]. The proposed mechanisms by which SGLT2 inhibitors improve these HF outcomes include intra‐ and extracellular volume reduction through glucosuria, natriuresis, and osmotic diuresis [5]. This could also explain the reduction in blood pressure and body weight, in addition to increased hematocrit and reduced preload [5]. Another proposed mechanism for cardiovascular protection in clinical studies is that SGLT2 inhibitors reduce afterload by improving endothelial function and reducing vascular stiffness [5, 6]. Furthermore, SGLT2 inhibitors moderately increase ketonemia, promoting a shift toward more energy‐efficient myocardial function [7, 8]. Aforementioned cardiovascular effects were described in healthy volunteers or patients who received long‐term SGLT2 inhibitor treatment in an out‐patient setting. The cardiovascular effects of short termSGLT2 inhibition during the perioperative period have not been studied.

The aim of this study was to investigate the cardiovascular effects of SGLT2 inhibition in patients undergoing cardiac surgery, as HF is a clinically relevant concern both pre‐ and postoperatively, and optimizing treatment with SGLT2 inhibitors could improve patient outcomes [9, 10]. We hypothesized that perioperative SGLT2 inhibition would be associated with increased diuresis leading to higher vasopressor requirements and greater intravenous fluid administration.

## 2 Materials and Methods

### 2.1 Trial Design

This is a sub‐study of the MERCURI trial, a single‐center open‐label randomized phase IV pilot study. The primary hypothesis of the MERCURI trial was that perioperative SGLT2 inhibition reduces postoperative AKI measured with the biomarker neutrophil gelatinase‐associated lipocalin (NGAL) in cardiac surgery patients. This study protocol was registered at the Dutch Trial Register (https://www.onderzoekmetmensen.nl/en/trial/52118) and was approved by the medical ethics committee of the Amsterdam UMC (ID: 2021_162) and by the Dutch competent authority before trial initiation. A detailed description of the MERCURI study, including changes in the protocol, and the primary outcomes is available open access [11]. The methodology described below pertains to the secondary analysis of hemodynamic outcomes in adherence to the CONSORT recommendations for reporting of randomized trials.

### 2.2 Study Participants

All participants from the intention‐to‐treat analysis of the MERCURI trial were included in this sub‐study. A detailed overview of all inclusion and exclusion criteria was previously published [11]. In summary, adult patients who were scheduled for elective cardiopulmonary bypass (CPB) assisted cardiac surgery were eligible. Exclusion criteria included type 1 diabetes (T1D), estimated glomerular filtration rate (eGFR) below 30 mL/min/1.73m^2^ and systolic blood pressure (SBP) below 100 mmHg. All participants signed informed consent before any trial‐related procedures.

### 2.3 Randomization and Blinding

Study participants were electronically randomized through the data management system Castor EDC (Ciwit BV, Amsterdam, the Netherlands). A balanced‐block randomization with random variable computer‐generated blocks of two, four, or six and an allocation ratio of 1:1 was used. Patients, care providers, and study personnel were not blinded as it was an open‐label study.

### 2.4 Intervention

Patients in the intervention group received an oral dose of 10 mg empagliflozin (Boehringer Ingelheim International GmbH, Ingelheim, Germany) every morning, starting 3 days before surgery, until 2 days after surgery. The control group received standard perioperative care (Figure S1). Apart from the intervention, patients were treated according to the discretion of the treating physicians.

### 2.5 Data Collection and Outcomes

Patient demographics, comorbidities, and kidney outcomes were recorded per study protocol, as previously reported [11]. For the analysis of our primary outcome, we recorded data on the hourly urine output, intravenous fluid administration, and calculated the fluid balance. All other data was collected to assess secondary outcomes, including hemoglobin (Hb) levels before surgery, at four time points on the day of surgery, and the morning of the first and second perioperative day; the number of patients that required allogeneic red blood cell transfusions; and the amount of red blood cell transfusions during hospitalization; the timing, dosages, and duration of norepinephrine, other vasoactive agents, and diuretics during the first 24 h postoperatively. Furthermore, SBP, diastolic blood pressure (DBP), mean arterial pressure (MAP), and heart rate (HR) were continuously measured in the Intensive Care Unit (ICU) postoperatively. Continuously measured variables were recorded as hourly means for analysis. We documented the incidence of postoperative atrial fibrillation (AF) by reviewing electronic health records of patients', as well as their routinely performed postoperative electrocardiograms (ECGs). AF de novo was defined as postoperative AF in patients with no known history of AF. Postoperative creatine kinase‐MB (CK‐MB) levels were routinely measured and recorded. The peak CK‐MB value was determined and used for between‐group comparison.

### 2.6 Statistical Analysis

We included all patients of the MERCURI trial in this analysis [11]. The MERCURI trial was powered to detect a significant between‐group difference in serum neutrophil gelatinase‐associated lipocalin (NGAL) levels [11]. Categorical variables are presented as counts with percentages per group and compared between groups with the chi‐square test or Fisher's exact test. Continuous variables are presented as mean ± SD and compared between groups using an unpaired Student's *t*‐test. Mean differences between groups with corresponding 95% confidence intervals (CIs) were calculated. Repeated measures such as blood pressure and HR were analyzed using a linear mixed‐effects model with time, group, and their interaction term as fixed effects, and subject as a random effect. The *p* value for the interaction term (time × intervention) was used to assess whether trends over time differed between treatment groups. All available repeated measures were included in the model under the assumption that data were missing at random; no imputation was performed. A Kaplan–Meier curve with the long‐rank test was used to assess whether there was a between‐group difference in the time required to wean norepinephrine. There was no correction for multiple testing. Statistical analyses were performed using SPSS (IBM version 24; IBM Corp., Armonk, New York) and GraphPad Prism (PRISM 10 for windows 64.0‐bit version 10.2.0, GraphPad Software LLC, Boston).

## 3 Results

This study included 55 participants; 25 were allocated to the intervention group and 30 to the control group. The baseline characteristics were well‐balanced (Table S1). Patients were predominantly male, with a mean age of 66 ± 10 years and underwent mostly valve repair/replacement or a combined surgery that included coronary artery bypass grafting (CABG). Preoperatively, patients demonstrated good kidney function (mean creatinine clearance 70 ± 16 mL/min), and the majority had a preserved left ventricular ejection fraction (> 50%).

### 3.1 Fluid Balance

**FIGURE 1:**
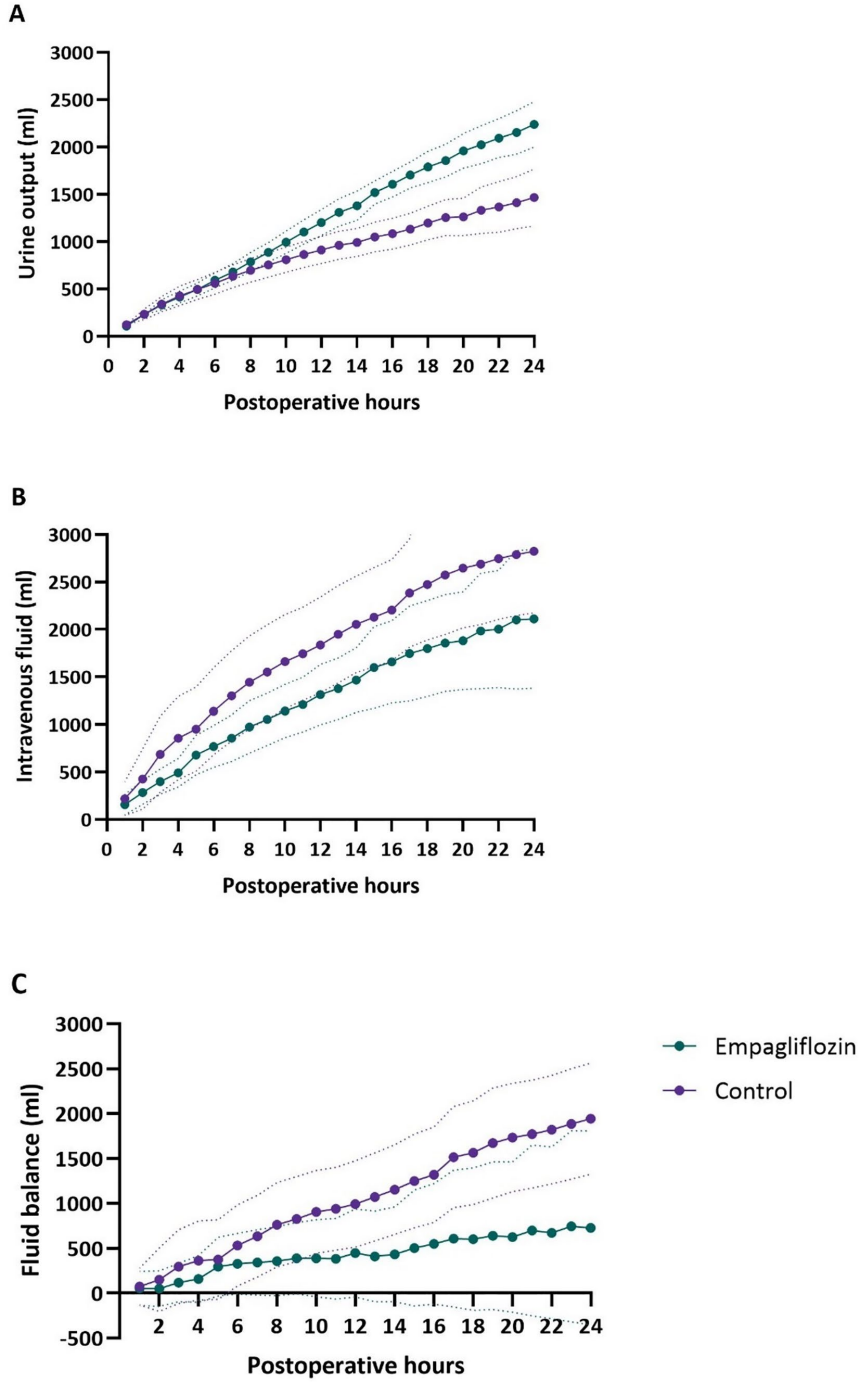
Overview of postoperative intravenous fluid administration, urine output and fluid balance. (A) Cumulative urine output. (B) Cumulative intravenous fluid suppletion. (C) Cumulative fluid balance.

The cumulative urine production during the first 24 h postoperatively was greater in the empagliflozin group: 1964 ± 542 mL compared to the control group: 1416 ± 529, mean difference 549 mL (95% CI 258–839, *p* < 0.001, Figure 1A). This was not associated with increased administration of intravenous fluids (empagliflozin 2110 ± 1730 mL vs. control 2823 ± 1739 mL, mean difference −712 mL, 95% CI, −1666—241, *p* = 0.140, Figure 1B). Taking these data together, this indicates a reduced positive fluid balance in the empagliflozin group: 726 ± 2554 mL compared to the control group: 1943 ± 1660, mean difference −1217 mL (95% CI −2373– −61, *p* = 0.039, Figure 1C). There was no difference in the number of patients receiving furosemide (empagliflozin: 6/25 vs. control: 8/30, *p* = 0.54) and no other diuretics were used.

**FIGURE 2:**
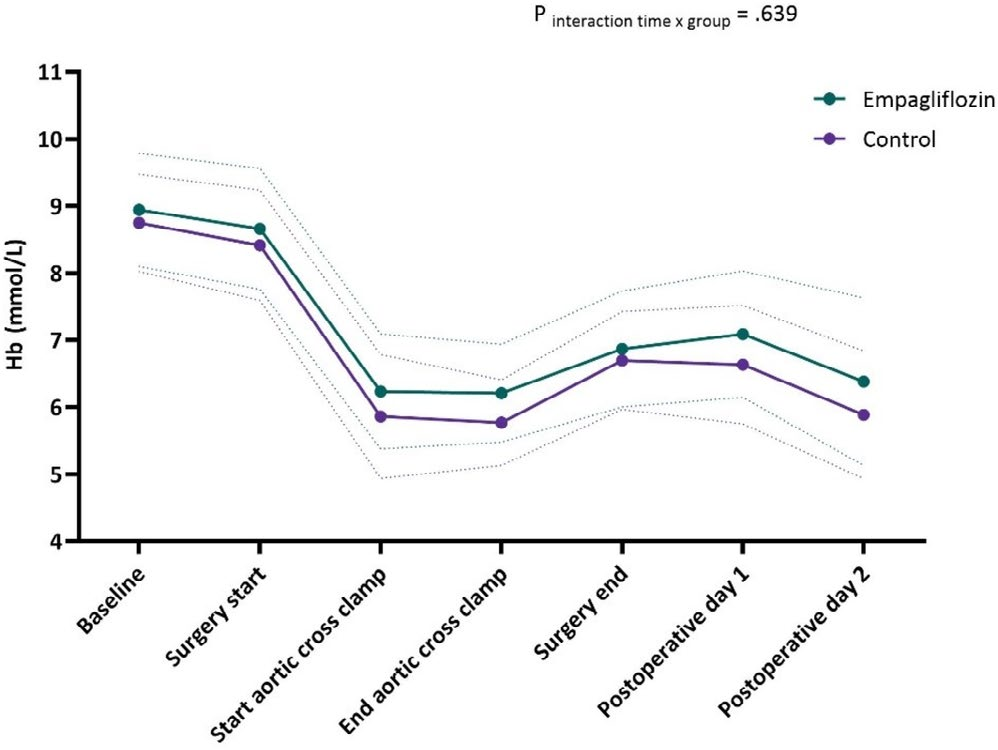
Perioperative hemoglobin levels. Hb = hemoglobin.

Perioperative Hb concentrations are presented in Figure 2. In the empagliflozin group, 6/25 patients received 3.5 ± 2.6 units of red blood cell transfusions postoperatively, compared to 13/30 patients in the control group, who received 2.6 ± 1.3 units (mean difference −0.9 [95% CI, −3.6–1.8, *p* = 0.456]).

### 3.2 Vasoactive Support

**FIGURE 3:**
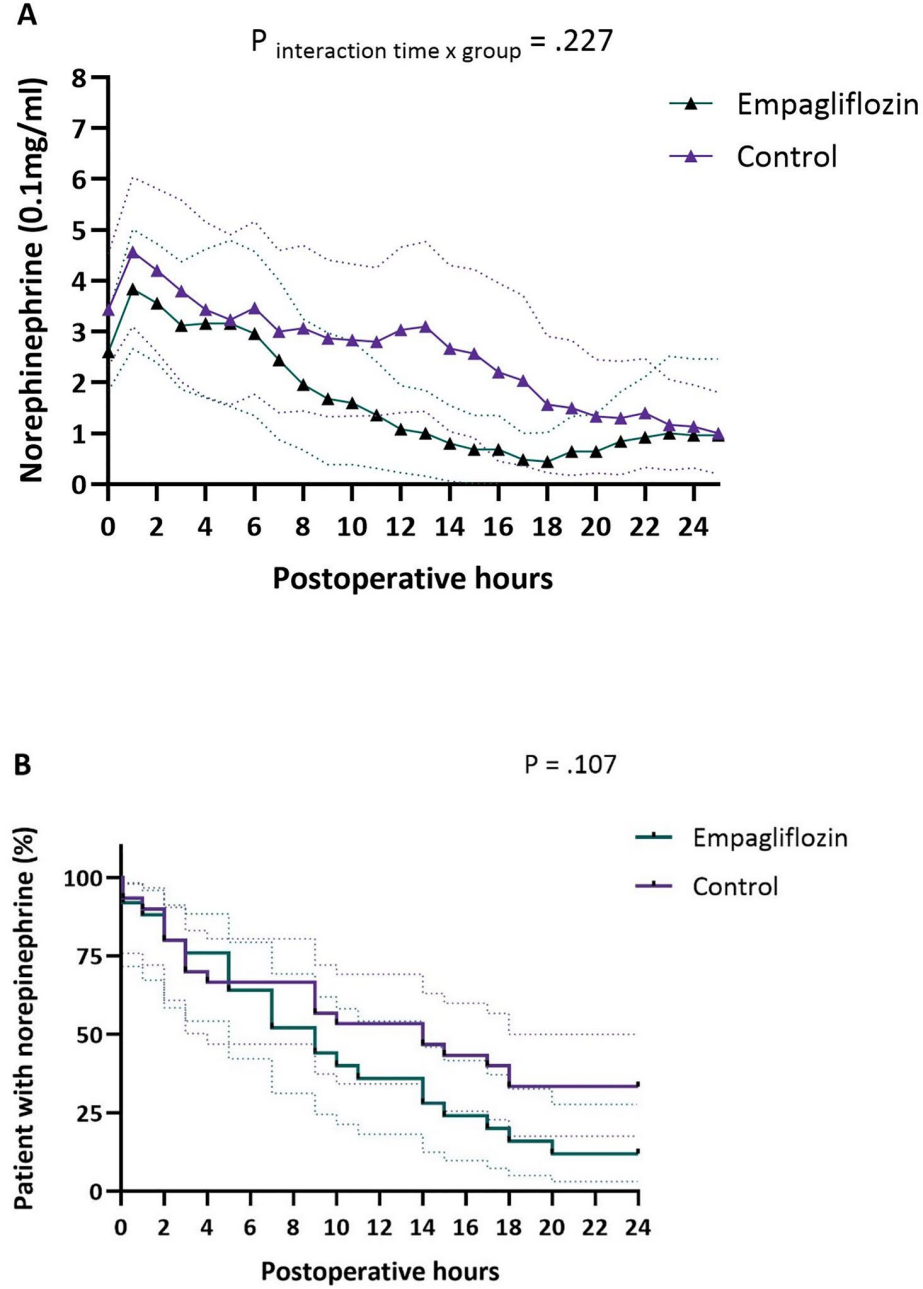
Postoperative norepinephrine use. (A) Mean dosage. (B) Time to wean off norepinephrine support.

Total dose of norepinephrine in the empagliflozin group was 4.1 ± 4.8 mg, compared to 6.2 ± 7.6 mg in the control group (difference 2.1 mg, 95% CI −1.4–5.6, *p* = 0.231). Norepinephrine administration during the first 24 h after surgery is visually presented in Figure 3A. The duration of norepinephrine support was 11.8 ± 11.5 h in the empagliflozin group compared to 19.3 ± 19.3 h in the control group (mean difference 7.5 h, 95% CI −1.3–16.4, *p* = 0.080). Figure 3B graphically depicts the time to wean off norepinephrine: it appeared that norepinephrine was weaned earlier in the empagliflozin group, but this failed to reach statistical significance (*p* = 0.107).

The use of other vasoactive agents was rare, and there were no differences between the groups; dobutamine (empagliflozin: 3/25 vs. control: 3/30, *p* = 1.000), milrinone (empagliflozin: 2/25 vs. control: 6/30, *p* = 0.269) or argipressin (empagliflozin: 0/25 vs. control: 2/30, *p* = 0.495).

### 3.3 Hemodynamic Parameters

**FIGURE 4:**
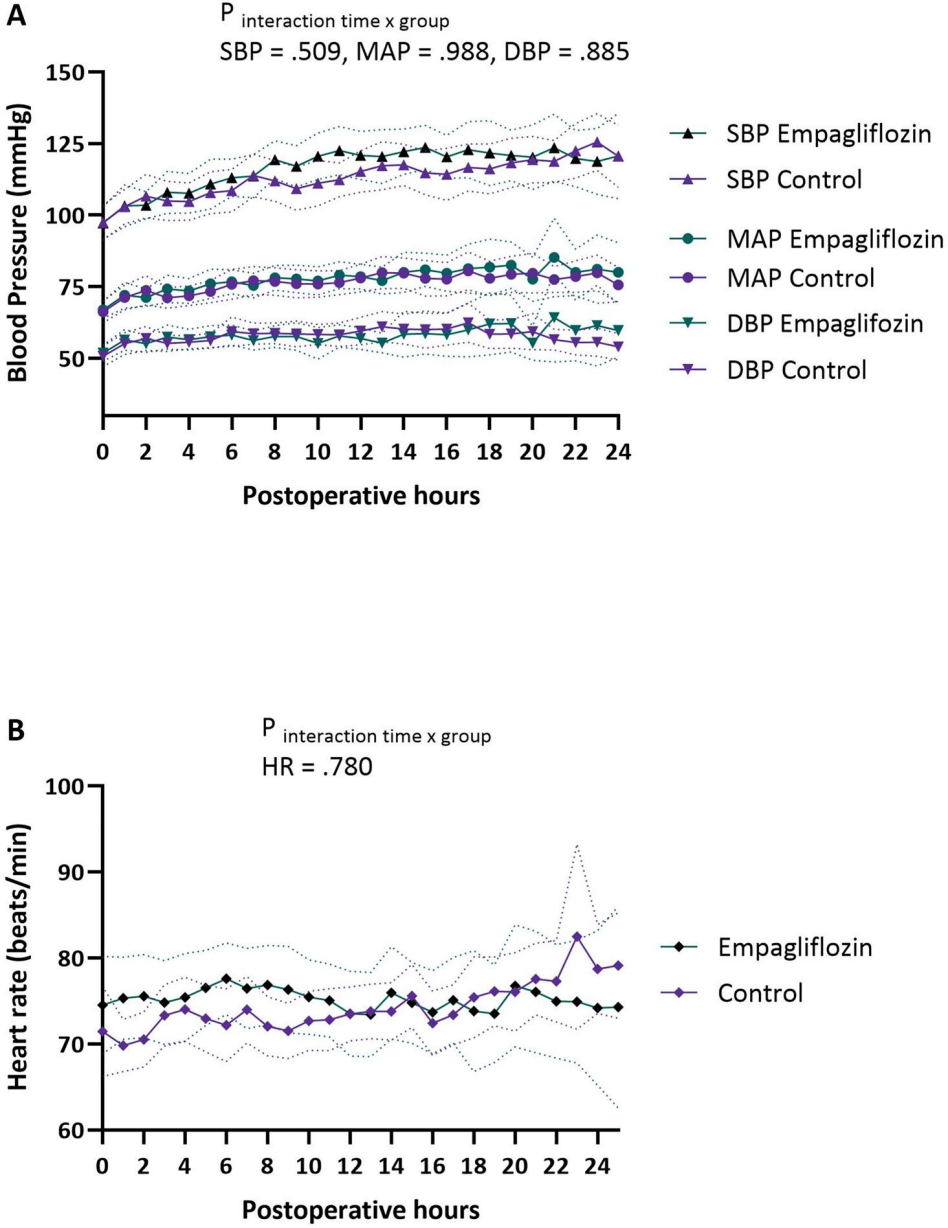
Postoperative hemodynamic measurements. (A) Blood pressure. (B) Heart rate.

Postoperative blood pressure and HR are visualized in Figure 4, and were not different between groups.

### 3.4 Atrial Fibrillation

Postoperative AF occurred in 13/25 patients in the empagliflozin group compared to 22/30 patients in the control group (*p* = 0.101). AF de novo was observed in 5/25 patients in the empagliflozin group compared to 10/30 patients in the control group (*p* = 0.269).

### 3.5 Cardiac Injury Biomarkers

There was no significant between‐group difference in peak CK‐MB, empagliflozin: 50.2 ± 47.7 μg/L vs. control: 63.8 ± 74.8 μg/L, with a mean difference of 13.6 μg/L (95% CI, −21.1–48.4, *p* = 0.435).

## 4 Discussion

In this randomized controlled trial, we observed that perioperative SGLT2 inhibition resulted in higher postoperative urine output, resulting in a more negative fluid balance. However, during the first 24 postoperative hours of ICU admission, we observed no between‐group differences in HR or blood pressure. The observed stabilization of vital signs was not achieved through more intravenous fluid supplementation or administration of vasoactive medication. Although not statistically significant, there was a trend toward a lower incidence of AF following SGLT2 inhibition. Despite the association between SGLT2 inhibitors and reduced blood pressure in other studies, [5] we observed no between‐group differences in blood pressure. We postulate that blood pressure in the intervention group was maintained through increased cardiac output, although this was not measured directly. Biological plausibility supporting this hypothesis includes that we observed no difference in HRs, while preload was presumably lower (as a result of more diuresis and less fluid administration), without compensation through increased systemic vascular resistance (no increase norepinephrine infusions). SGLT2 inhibitors were found to decrease vascular resistance, but SGLT2 inhibitor treated patients in this study needed less vasopressor support [7, 12]. The proposed improvement in cardiac output is supported by previous studies showing that SGLT2 inhibitors improve cardiac function through improved diastolic function [13, 14, 15, 16]. In patients with type 2 diabetes who received SGLT2 inhibitors for 6 months, left ventricular mass index and left atrial volume index, determined by echocardiography, significantly decreased [14]. In the current study, echocardiography was not routinely available; it would be interesting to evaluate the effect of short‐term SGLT2 inhibition on diastolic function in patients undergoing cardiac surgery.

We observed increased diuresis after SGLT2 inhibition compared to the control group. The diuretic effect of SGLT2 inhibitors remains a subject of ongoing debate. Some studies have reported increased urine output, while other studies have reported no significant diuretic effect [17, 18]. A study that measured 24 h urine volumes in patients with chronic kidney disease on a standardized sodium diet treated with SGLT2 inhibitors observed no increase in diuresis and concluded that the activation of compensatory mechanisms within the kidney prevents increased diuresis [17]. The postoperative cardiac surgery setting of our study is different from studies involving stable outpatients. The higher urine output observed in our study may indicate preserved kidney function compared to the control group, where we observed oliguria.

The incidence of de novo postoperative AF was lower in the group receiving SGLT2 inhibitors; however, this was not significant. The study population was too small to detect a significant difference in the incidence of de novo postoperative AF. Given that SGLT2 inhibitors are associated with reduced preload, afterload, atrial size, and calcium overload, it would not be surprising to find that SGLT2 inhibitors decrease the incidence of postoperative AF in patients undergoing cardiac surgery; [5, 19, 20, 21] an association that has already been shown in large meta‐analyses with patients on chronic SGLT2 inhibition treatment [22, 23]. It would be interesting to investigate whether SLGT2 inhibitors lower the incidence of postoperative AF following cardiac surgery, as AF is a common postoperative complication in this patient population and is associated with heart failure, cerebrovascular events, and increased health care costs [22]. Similarly, while the difference in the duration of norepinephrine support did not reach statistical significance, it remains an important finding that merits further evaluation in larger trials.

Despite the small single center nature of this study, we can highlight the following strengths. We recorded real‐world data from routine clinical care; fluids and vasopressors were administered according to Intensive Care physician and nurse driven protocols. Analyses for this substudy included all participants without selection bias or missing data. Nonetheless, we note several limitations: this was an open‐label study, so by design, there was no blinding. This may have introduced bias in clinical decision‐making; however, all patients in this single center trial were treated according to the same in‐hospital protocols. We lacked a form of continuous cardiac output monitoring or timely postoperative echocardiography. Cardiovascular outcomes were included in the secondary outcome of this study; hence, all results are considered hypothesis‐generating only. However, the fact that all significant findings fit within the presumed physiological explanation and are in line with previous research findings reduces the likelihood that these results were based by chance only. Results warrant further investigation, and we are currently conducting a large, multi‐center follow‐up trial with postoperative AF as a predefined secondary outcome. We initiated perioperative treatment with SGLT2 inhibitors in contrast to the current guidelines. Further research is warranted to assess whether these recommendations should be updated.

## 5 Conclusion

In this study, perioperative SGLT2 inhibition was associated with increased diuresis and lesser fluid accumulation without an increase in vasopressor requirements. SGLT2 inhibition was not associated with an increased risk of AKI, hypotension, or arrhythmias.

## Author Contributions

**Lars I. P. Snel:** investigation, formal analysis, validation, writing – original draft. **Maartina J. P. Oosterom‐Eijmael:** investigation, formal analysis, validation, writing – original draft. **Yugeesh R. Lankadeva:** writing – review and editing, supervision. **Mark P. Plummer:** writing – review and editing. **Benedikt Preckel:** resources, writing – review and editing, supervision. **Coert J. Zuurbier:** writing – review and editing. **Daniel H. van Raalte:** conceptualization, methodology, writing – review and editing, supervision. **Jeroen Hermanides:** conceptualization, methodology, writing – review and editing, supervision. **Abraham H. Hulst:** conceptualization, methodology, writing – review and editing, supervision, funding acquisition.

## Conflicts of Interest

D.H.v.R. has served as a consultant and received honoraria from Boehringer Ingelheim and Lilly, Merck, Sanofi, and AstraZeneca and has received research operating funds from Boehringer Ingelheim and Lilly Diabetes Alliance and AstraZeneca; all honoraria are paid to his employer (Amsterdam University Medical Center, Amsterdam). The other authors declare no conflicts of interest.

## Supporting information


Data S1.

Data S2.

## Data Availability

The data that support the findings of this study are available from the corresponding author upon reasonable request.
