# Supplementary material for: Cardiovascular Outcomes of Perioperative Sodium‐Glucose Transporter 2 Inhibition in Cardiac Surgery Patients: An Open‐Label Randomized Pilot Study
Source: Acta Anaesthesiol Scand. 2025 Jun 22;69(6):e70075. doi: 10.1111/aas.70075 (PMC12182971; doi:10.1111/aas.70075)
Supplement: Supplementary file 1 — Data S1. [file AAS-69-0-s002.docx]

**Supplementary Tables and Figures**

Supplementary Table 1.

Baseline characteristics of the intention-to-treat population.

|  | **All**  **(n=55)** | **Empagliflozin**  **(n=25)** | **Control**  **(n=30)** |
| --- | --- | --- | --- |
| Age, mean ± SD, years | 66 ± 10 | 69 ± 8 | 63 ± 10 |
| Male sex, n (%) | 40 (73) | 18 (72) | 22 (73) |
| Ethnic origin, n (%) |  |  |  |
| Caucasian | 53 (96) | 24 (96) | 29 (97) |
| Other | 2 (4) | 1 (4) | 1 (3) |
| BMI, mean ± SD, kg/m² | 28.0 ± 4.4 | 27.6 ± 3.4 | 28.2 ±5.1 |
| T2D, n (%) | 5 (9) | 3 (12) | 2 (7) |
| Diabetes medication, n (%) |  |  |  |
| Metformin | 4 (7) | 3 (12) | 1 (3) |
| Sulfonylurea derivate | 1 (2) | 0 (0) | 1 (3) |
| Hypertension, n (%) | 28 (51) | 13 (52) | 15 (50) |
| NYHA classification, n (%) |  |  |  |
| I | 18 (33) | 10 (40) | 8 (27) |
| II | 28 (51) | 12 (48) | 16 (53) |
| III | 9 (16) | 3 (12) | 6 (20) |
| LVEF, n (%) |  |  |  |
| Good > 50% | 40 (73) | 20 (80) | 20 (67) |
| Moderate 31-50% | 15 (27) | 5 (20) | 10 (33) |
| ASA physical status classification, n (%) |  |  |  |
| I | 1 (2) | 0 (0) | 1 (3) |
| II | 7 (13) | 2 (8) | 5 (17) |
| III | 45 (82) | 22 (88) | 23 (77) |
| IV | 2 (4) | 1 (4) | 1 (3) |
| Creatinine clearance, mean ± SD, ml/min | 70 ± 16 | 68 ± 16 | 73 ±15 |
| EuroSCORE II, mean ± SD, % | 3.0 ± 3.2 | 2.8 ±3.4 | 3.1 ± 3.0 |
| Duration of surgery, mean ± SD, min | 271 ± 96 | 249 ± 83 | 291 ± 104 |
| Type of surgery, n (%) |  |  |  |
| CABG procedure | 3 (6) | 1(4) | 2 (7) |
| Single non-CABG procedure | 28 (51) | 12 (48) | 16 (53) |
| Two or more procedures | 24 (44) | 12 (48) | 12 (40) |
| Intraoperative fluid suppletion, mean ± SD, L | 3.4 ± 1.3 | 3.2 ± 1.2 | 3.5 ± 1.4 |

|  |
| --- |

*ASA= American Society of Anesthesiologists; BMI= body mass index; CABG= coronary artery bypass graft; EuroSCORE= European System for Cardiac Operative Risk Evaluation; IQR= interquartile range; LVEF= left ventricular ejection fraction; NYHA= New York Heart Association; SD= standard deviation; T2D= type 2 diabetes*

Supplementary Figure 1.

Summary of study workflow.

*
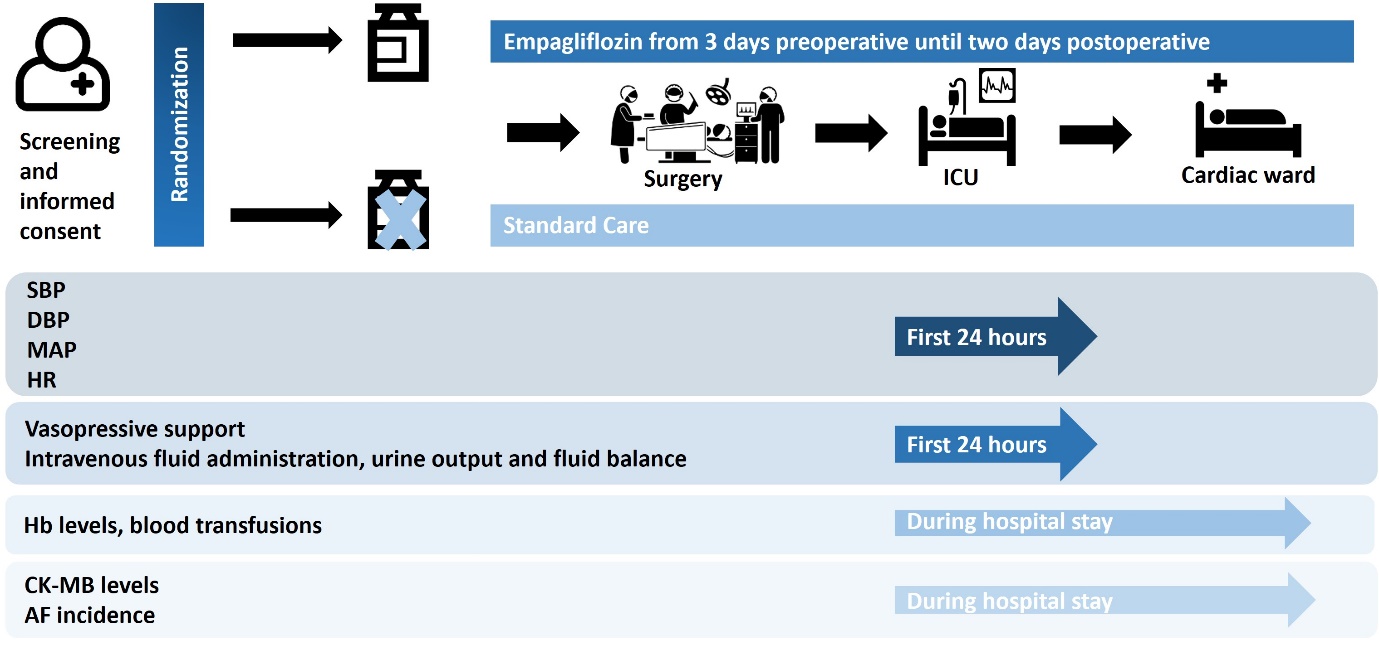
*

*AF = arterial fibrillation; CK-MB = creatinine kinase-MB; DBP = diastolic blood pressure; HB = hemoglobin; HR = heart rate; ICU = Intensive Care Unit; MAP = mean arterial pressure; SBP = systolic blood pressure;*
